# Transcriptional Analysis of Temporal Gene Expression in Germinating *Clostridium difficile* 630 Endospores

**DOI:** 10.1371/journal.pone.0064011

**Published:** 2013-05-15

**Authors:** Marcin Dembek, Richard A. Stabler, Adam A. Witney, Brendan W. Wren, Neil F. Fairweather

**Affiliations:** 1 MRC Centre for Molecular Bacteriology and Infection, Department of Life Sciences, Imperial College London, London, United Kingdom; 2 Faculty of Infectious and Tropical Diseases, London School of Hygiene and Tropical Medicine, London, United Kingdom; 3 Division of Clinical Sciences, St George’s, University of London, London, United Kingdom; University of Liverpool, United Kingdom

## Abstract

*Clostridium difficile* is the leading cause of hospital acquired diarrhoea in industrialised countries. Under conditions that are not favourable for growth, the pathogen produces metabolically dormant endospores *via* asymmetric cell division. These are extremely resistant to both chemical and physical stress and provide the mechanism by which *C. difficile* can evade the potentially fatal consequences of exposure to heat, oxygen, alcohol, and certain disinfectants. Spores are the primary infective agent and must germinate to allow for vegetative cell growth and toxin production. While spore germination in *Bacillus* is well understood, little is known about *C. difficile* germination and outgrowth. Here we use genome-wide transcriptional analysis to elucidate the temporal gene expression patterns in *C. difficile* 630 endospore germination. We have optimized methods for large scale production and purification of spores. The germination characteristics of purified spores have been characterized and RNA extraction protocols have been optimized. Gene expression was highly dynamic during germination and outgrowth, and was found to involve a large number of genes. Using this genome-wide, microarray approach we have identified 511 genes that are significantly up- or down-regulated during *C. difficile* germination (*p*≤0.01). A number of functional groups of genes appeared to be co-regulated. These included transport, protein synthesis and secretion, motility and chemotaxis as well as cell wall biogenesis. These data give insight into how *C. difficile* re-establishes its metabolism, re-builds the basic structures of the vegetative cell and resumes growth.

## Introduction

*Clostridium difficile* is a Gram-positive, anaerobic bacterium and a leading cause of antibiotic-associated diarrhoea in industrialised countries [1]. Infection typically occurs among hospitalized patients, whose natural intestinal microflora has been disrupted by prolonged treatment with broad-spectrum antibiotics, allowing the pathogen to colonize the compromised gastro-intestinal tract [2]. The resulting, toxin-mediated disease can range from mild, self-limiting diarrhoea through severe diarrhoea to the potentially lethal pseudomembraneus colitis and can progress to toxic megacolon and sepsis syndrome causing significant morbidity and mortality [3].

Under conditions that are not favourable for growth, *C. difficile* exits the vegetative growth cycle and triggers sporulation, producing metabolically dormant endospores (spores). These are thought to be the primary infectious agent as recent studies have shown that a mutant strain of *C. difficile* unable to produce SpoOA, a regulatory protein essential for spore formation, is unable to efficiently persist and transmit the disease [4]. Due to their multi-layered structure, spores are extremely robust and resistant to both chemical and physical insult, providing the mechanism by which *C. difficile* can evade the potentially fatal consequences of exposure to heat, oxygen, alcohol, and certain disinfectants [reviewed in 5,6]. Spores shed in faeces are therefore difficult to eradicate and can persist in healthcare facilities for extended periods of time leading to infection or re-infection of individuals through inadvertent ingestion of contaminated material [7], [8].

In order to cause disease, spores need to return to vegetative growth through a process termed germination. In *Bacillus*, *Clostridium* and related species, spore germination is initiated upon binding of small molecules called germinants (often nutrients such as sugars and/or amino acids) to specific germination receptors (GRs). This triggers a series of irreversible biophysical events that lead to rehydration of the spore core and degradation of its protective layers. Once the constraints of spore outer layers are lifted, the cell enters a period of longitudinal growth, accompanied by re-establishment of cell metabolism during which DNA, RNA and protein synthesis resume [reviewed in 9]. While spore germination in *Bacillus* is well understood, and genes involved in this process have recently been identified in *Clostridium perfringens* [10]–[12], little is known about *C. difficile* germination and outgrowth. Even though many components of the spore germination machinery are conserved between spore forming members of *Bacillales* and *Clostridiales*, recent studies have revealed significant differences both in the proteins and in the signal transduction pathways involved [reviewed in 13]. Bioinformatic analysis of *C. difficile* genome has failed to reveal genes encoding known germination receptor subunits [14]–[16] even though a number of such genes were identified in other Clostridia. Furthermore, while the *C. difficile* 630 spore proteome has recently been described [17], little homology has been found between *C. difficile* spore proteins and those present in other spore-formers. This would suggest that the aspects of germination in *C. difficile* are unique, a notion supported by the limited numbers of studies published so far [18]–[21].

We now know that bile salts (cholate, taurocholate, glycocholate and deoxycholate) stimulate *C. difficile* spore germination [21]. More recently glycine and histidine were shown to act as a co-germinants with these cholate derivatives [18], [22], [23], and kinetic studies suggest that there are distinct germination receptors for taurocholate and glycine [24]. Neither of these compounds has been previously described as a germinant for spores of *Bacillus* or *Clostridium* species, supporting the notion of a novel mode of germinant recognition in *C. difficile* spores. Furthermore, chenodeoxycholate, another bile salt, has been shown to inhibit *C. difficile* spore germination [19], adding a new level of regulation to the current model of *C. difficile* colonisation of the gut.

As limited as our insight into the mechanism of germination in *C. difficile* might be, even less is known about the events that follow initiation of germination and while transcriptomic analysis of gene expression during germination has been carried out in *B. subtilis* [*25*] and more recently *C. novyi-NT* [26] and *C. sporogenes* [*27*], no such data is available for *C. difficile.* To address this issue we use a combination of standard microbiology, microscopy and genome-wide transcriptome analysis to explore the morphological, physiological and transcriptional changes that occur during germination and subsequent outgrowth of *C. difficile* 630 spores and try to elucidate some of the processes that occur during the transformation of a metabolically dormant spore into an actively growing vegetative cell.

## Results and Discussion

### Characterization of Germination and Outgrowth Dynamics

**Figure 1:**
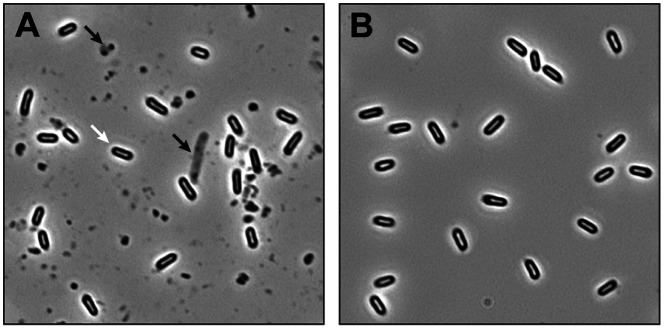
*C. difficile* spore purification. *C. difficile* was sporulated on SMC solid medium, washed extensively and purified *via* density gradient centrifugation. Samples taken before and after purification were analysed using phase contrast microscopy A) Crude spore preparation before purification. Phase-bright endospores (white arrow), vegetative cells and significant amount of cell debris (black arrows) are visible**.** B) Spore preparation after purification. Phase-bright endospores are visible, free of vegetative cells and cell debris.

*C. difficile* 630, an epidemic, virulent and multi-drug-resistant strain was selected for this analysis, as its complete genome sequence has been determined [15]. In order to obtain sufficient quantities of spores, free from vegetative cells and cell debris, a protocol for producing and purifying spores was developed. Growth of *C. difficile* 630 on solid SMC sporulation medium for 7 days ensured the highest sporulation rates (data not shown). Using these growth conditions, 10^7^ - 10^8^ endospores could be reproducibly obtained per ml of initial culture. Phase contrast microscopy confirmed the high purity of the purified spore suspensions, revealing fully developed, phase-bright endospores, free of vegetative cells and noticeable cell debris (Figure 1).

**Figure 2:**
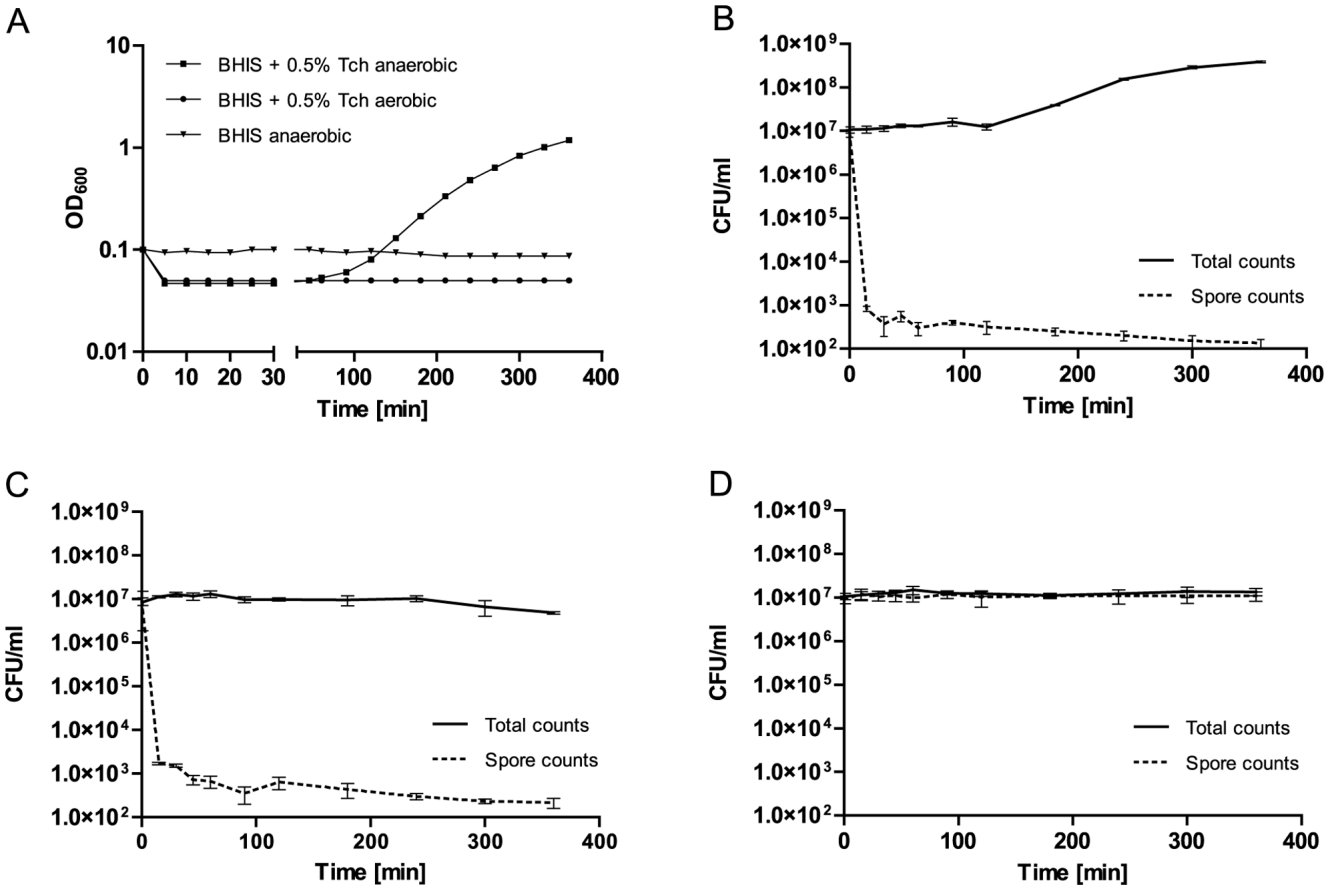
Growth characteristics during spore germination and outgrowth. Purified *C. difficile* 630 endospores were resuspended in BHIS ±0.5% sodium taurocholate (Tch) to an OD_600_ of 0.1 and incubated either aerobically or anaerobically for 6 h at 37°C. Growth was monitored *via* OD_600_ measurements and CFU counts. A) *C. difficile* 630 germination dynamics growth curve. Spores incubated in the presence of 0.5% Tch showed a rapid decrease in OD_600_ immediately after resuspension, in both aerobic and anaerobic conditions. No decrease in OD_600_ was observed for spores incubated in the absence of Tch. B) CFU counts for spores germinated anaerobically in BHIS +0.5% Tch. C) CFU counts for spores germinated aerobically in BHIS +0.5% Tch. D) CFU counts for spores germinated anaerobically in BHIS. Data reported as means and standard deviations from three independent experiments.

**Figure 3:**
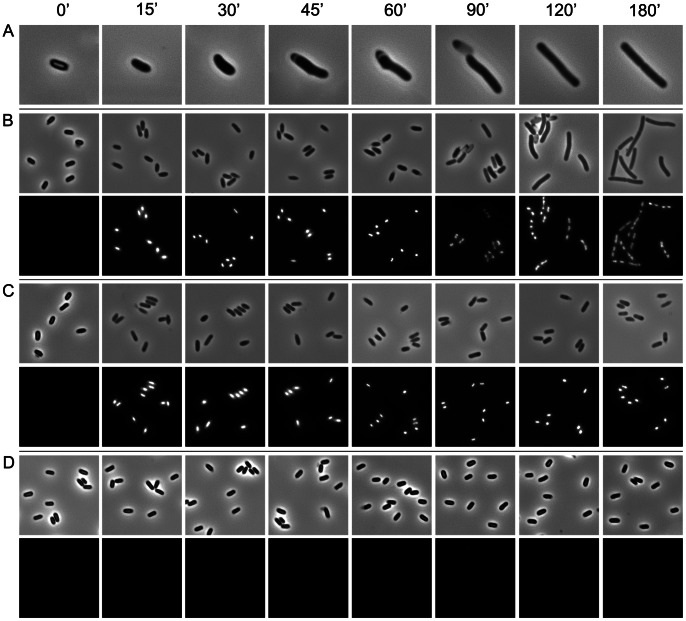
Morphological changes during spore germination and outgrowth. Purified *C. difficile* 630 endospores were resuspended in BHIS ±0.5% sodium taurocholate (Tch) to an OD_600_ of 0.1 and incubated either aerobically or anaerobically for 6 h at 37°C. At the given time points samples were formaldehyde-fixed and analysed using phase contrast microscopy and fluorescence microscopy following DNA staining with Hoechst 33258. A and B) Spores germinated anaerobically in BHIS +0.5% Tch. Upon induction of germination, dormant spores lose their phase-bright appearance and become susceptible to DNA staining. This is followed by a gradual increase in volume and shedding of spore outer layers which remain visible as empty ‘shells’. Once the physical constraints of the outer spore layers are removed, the spore enters a phase of longitudinal growth coinciding with DNA replication followed by symmetric cell division. C) Spores germinated aerobically in BHIS +0.5% Tch. Following induction of germination, spores lose their phase-bright appearance and become susceptible to DNA staining but fail to progress to outgrowth. D) Spores germinated anaerobically in BHIS. No changes in spore appearance and no DNA staining could be observed.

To ensure that the purification process does not detrimentally affect the spores, the viability and germination dynamics of the isolated spores were assessed. Spore germination is classically measured as a decrease in optical density (OD) of a spore suspension occurring concomitantly with the release of dipicolinic acid (DPA) from the spore core, rehydration of the core, and degradation of the cortex [9]. This is followed by an increase in OD_600_ correlated with outgrowth and cell division as the cells enter logarithmic growth phase. OD_600_ measurement of liquid cultures containing germinating spores combined with colony forming unit (CFU) counts and microscopy showed that >99.9999% of spores undergo germination when grown in nutrient medium (BHIS) supplemented with the germinant, sodium taurocholate (Tch) as indicated by a rapid decrease in OD_600_ (approx. 50% of initial value within 5 minutes of induction) and a 5-log drop in the number of spore CFUs (Figure 2A, 2B and 2C). Importantly, based on phase contrast and fluorescence microscopy analysis, spore germination in nutrient medium supplemented with an excess of Tch was synchronous and appeared to be complete within 180 min. This would be critical in subsequent transcriptomic analysis of gene expression during germination as it ensured that all spores were in the same phase of germination. Immediately upon induction of germination, the dormant spores lost their phase-bright appearance becoming susceptible to Hoechst 33258 DNA staining, presumably due to Ca^2+^-DPA release and subsequent rehydration of the spore core resulting in a gradual increase in spore volume (0 - 30 min). This was followed by hydrolysis of cortex peptidoglycan and shedding of spore outer layers (45–60 min). Once these physical constraints were removed, germinating cells entered a phase of longitudinal growth which coincided with DNA replication followed by symmetric cell division (60–180 min) (Figure 3A and 3B). In contrast, no germination was observed among spores grown in medium devoid of the germinant as no changes in OD_600_, spore CFU count or spore morphology could be observed throughout the 6 h incubation period (Figure 2A and 2D; Figure 3D). Unlike in *Bacillus,* where pre-treating spores with high temperature increases germination rates, we found that heat activation had no effect on germination dynamics of the isolated *C. difficile* endospores (data not shown). Interestingly, germination was not observed when liquid cultures were incubated aerobically, presumably due to the inhibitory presence of oxygen. Even though a decrease in OD_600_ and spore CFUs was observed immediately after induction with Tch and the spores lost their phase-bright appearance becoming susceptible to DNA staining (indicative of Ca^2+^-DPA release and core rehydration), spores failed to progress fully to outgrowth and eventually were killed as indicated by the gradual drop in total CFUs (Figure 2A and 2C; Figure 3C). This is consistent with recent findings [28] and suggests that oxygen is a negative regulator of *C. difficile* endospore germination acting downstream of any signalling events that induce germination.

### Optimization of RNA Extraction

RNA quality is of paramount importance in any transcriptional analysis of gene expression. It was therefore critical to develop a method for extracting RNA that would not only provide high yields, but would also ensure that the integrity of RNA was maintained. Mechanical disruption with silica beads followed by acid phenol-based extraction was shown to give the highest quality RNA. Using this method, between 2 and 10 µg of total RNA could be reproducibly isolated per 10^8^ spores. The quantity of extracted RNA depended on the status of the spores and increased gradually as the spores went from dormancy through germination to outgrowth (Figure S1A). This could be attributed to the increasing susceptibility of the spore envelope to lysis during germination and/or the initiation of RNA synthesis [9]. All preparations were of high purity and integrity as indicated by a 260/280 nm absorption ratio in the range of 2.05 to 2.18 and Bioanalyzer electropherograms showing sharp and undegraded peaks (Figure S1B). Interestingly, we found that the ribosomal RNA (rRNA) of spores differed from that present in vegetative cells. In addition to the 16S and 23S rRNA species, two smaller rRNA species were identified in dormant spores, represented by small peaks on Bioanalyzer spectra (Figure S1C). These could only be discerned in dormant spores and reduced in intensity as germination progressed. A similar phenomenon has previously been reported in *Bacillus* [25] as well as the more closely related *Clostridium novyi* [26] and *Clostridium sporogenes* [27], although its significance remains unknown. These smaller species might represent rRNA fragments or be indicative of RNA maturation within the germinating spore [29].

### Microarray Analysis of Temporal Gene Expression during Spore Germination – General Observations

**Figure 4:**
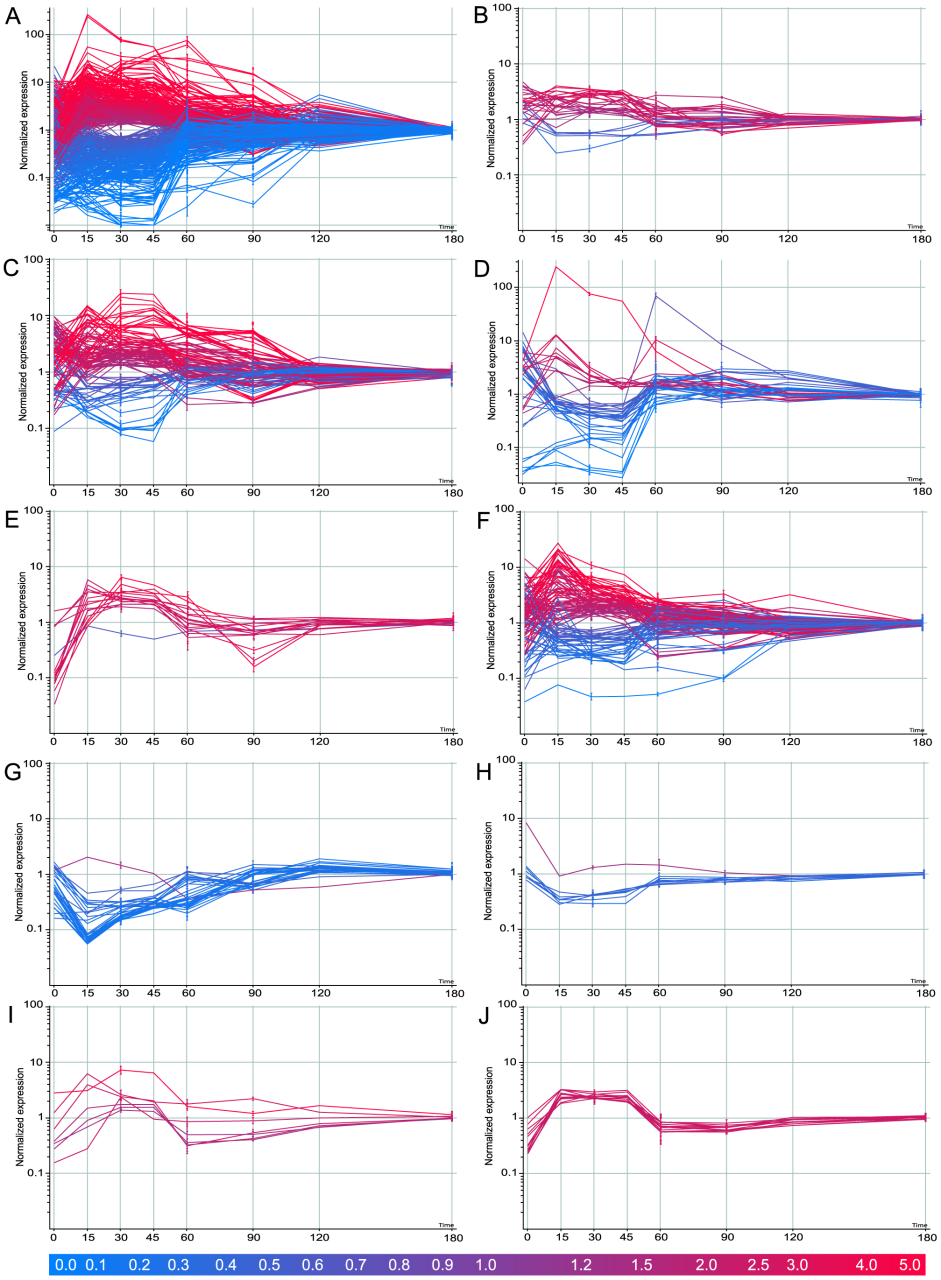
Temporal gene expression profiles in germinating *C. difficile* spores. A) All significantly, differentially regulated genes, B) two-component systems, C) ABC-transporters, D) phosphotransferase system, E) ribosomal proteins, F) transcriptional regulators, G) flagellar assembly and chemotaxis, H) type IV pili, I) peptidoglycan biosynthesis, J) secondary cell wall polymer (SCWP) biosynthesis are shown. Microarray gene expression data represented as normalised intensity with respect to control conditions. Analysed using 1-way ANOVA (A *p*≤0.01, B-J *p*≤0.05) and Benjamini-Hochberg multiple testing correction. Each line represents data for one gene. All graphs were generated using GeneSpring GX7.3.1.

**Figure 5:**
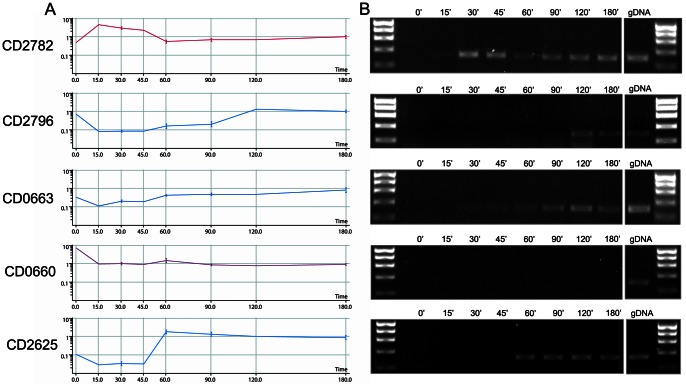
RT-PCR validation of microarray results. cDNA was prepared from total RNA using reverse transcription. Primers internal to five selected genes were used to amplify fragments of ∼150 bp: (CD2782 - cell wall binding protein, CD2796 - cell wall binding protein, CD0663 - toxin A, CD0660 - toxin B, CD2625 - putative membrane protein). gDNA was used as template in a positive control reaction. 25 reaction cycles were carried out to ensure non-saturating conditions. A) microarray expression profile (normalised to 180 minutes), B) gel electrophoresis of the PCR amplicons.

Total RNA extracted from germinating spores at eight time points representing dormancy (0 min), germination (15, 30, 45, 60 min) and outgrowth (90, 120, 180 min) was analysed by competitive RNA/DNA hybridisation using the BµG@S CDv2.0.1 microarray. Gene expression was highly dynamic during germination and outgrowth and was found to involve a large part of the genome. Relatively few transcripts were identified in dormant spores (see below), consistent with their metabolically silent state. In contrast, a significant up-regulation of gene expression was observed immediately after induction of germination, peaking at 30 min (Figure 4). In order to validate these results, semi-quantitative RT PCR was used to confirm the temporal expression pattern for a number of selected genes showing a wide range of distinct expression profiles being either significantly up- or down-regulated in the early or late stage of germination. In all cases the expression profiles obtained matched those seen in the microarray experiment (Figure 5).

We investigated in detail two time points: 30 min and 180 min post germination. At 30 min all spores are actively germinating in contrast to 180 min when normal vegetative growth has commenced. In total, 263 genes were up-regulated and 248 genes down-regulated at 30 min when compared to the 180 min time point (*p*≤0.01) (Figure 4A). The magnitude of change of the statistically up-regulated genes ranged from 1.3-fold to 80-fold, and that of down-regulated genes ranged from 1.3- to 100-fold. Many gene clusters encoding enzyme complexes or biochemical pathways appeared to be co-ordinately regulated. The Kyoto Encyclopedia of Genes and Genomes (KEGG) database was used to identify significantly differentially regulated pathways. Selected examples have been listed below and summarised in Table 1. Where appropriate, genes that marginally failed the stringent statistical cut-off (0.01≤*p*≤0.05) are also mentioned. Full lists of genes generated through this experiment are shown in supplementary data (Table S1).

**Table 1:** Numbers of genes differentially regulated during germination.

Functional group	Up-regulated	Down-regulated
Two-component systems	26	5
ABC-transporters	71	34
Phosphotransferase system	11	30
Ribosomal proteins	12	1
Transcriptional regulators	45	24
Flagellum and chemotaxis	0	34
Type IV pili	0	7
Peptidoglycan biosynthesis	9	1
Secondary cell wallpolymers	12	0

The total number of genes within specific functional groups up-regulated or down-regulated at 30 min into germination was determined using microarray analysis (***p*** **≤0.05)**.pone.0064011.g006.tif.

### Spore Transcripts

Despite early research hinting at the presence of mRNA in spores [30], [31], for a long time it has been generally assumed that dormant spores do not contain functional transcripts. This view has now changed as recent studies conducted in *B. subtilis* [25], *C. novyi-NT* [26] and *C. sporogenes* [27] have shown that mRNA is abundant in spores and that its composition is dramatically different from that observed in vegetative cells. A number of explanations for this phenomenon have been proposed. Spore transcripts might represent mRNA that has been entrapped during the late phases of sporulation and is later degraded to act as a reservoir of nucleotides during germination. Indeed, five out of fifteen most abundant spore transcripts identified in this study represented late-sporulation genes such as those encoding small acid-soluble proteins A and B (CD2688 and CD3249 respectively) a putative spore coat protein (CD0213) and a stage IV sporulation protein (CD0783) (Table S2). Alternatively, transcripts might be stored to equip the spore with proteins that will become necessary during the transformation into a vegetative cell such as those involved in growth, protein synthesis, detoxification, metabolism, transport, secretion etc. Interestingly, the second most abundant transcript we have identified encodes a putative Mn-superoxide dismutase (CD1631). Other genes with redox activity whose transcripts were present at relatively high levels in dormant spores included two putative oxidoreductase complexes with ferrodoxin activity (CD2197-CD2199A and CD2427-CD2429A), a NADH oxidase (CD2540) and a NADH-dependent flavin oxidoreductase (CD2709). The inclusion of such a large number of redox genes within the spore transcriptome is intriguing, particularly in the light of the anaerobic life cycle of *C. difficile*. While *C. difficile* spores are resistant to ambient oxygen concentrations, vegetative cells can be killed by relatively short exposure to oxygen. It would thus be tempting to speculate that at least a subset of these genes might play a role in detoxification by scavenging reactive oxygen species (ROS). Finally, consistent with previous reports [26], we found that a large proportion of spore transcripts encode proteins with an unknown function (nine out of top fifteen hits). This not only underlines the difference in mRNA composition between spores and vegetative cells but also the need for more research into the spore transcriptome of major spore-forming bacteria.

### Two-component Signal Transduction Systems

Two-component regulatory systems serve as a basic stimulus-response coupling mechanism to allow organisms to sense and respond to changes in many different environmental conditions. They typically consist of a membrane-bound histidine kinase that senses a specific environmental stimulus and a corresponding response regulator that mediates the cellular response, mostly through differential expression of target genes [reviewed in 32]. *C. difficile* 630 has fifty-one two-component sensor histidine kinases and fifty-four response regulators. Nine kinase genes and seventeen response regulator genes were up-regulated in germinating spores. Only five genes (three kinases and two response regulators) were down-regulated (Figure 4B).

### Transport of Metabolites and Sugars

The germinating spore requires a large supply of metabolites and cofactors to facilitate re-establishment of cell metabolism. Consistent with this requirement, we found 100 out of 229 ABC-transporter genes were differentially regulated during germination. A majority of these (70 genes) were up-regulated during germination when compared to the vegetative state, including the entire *potABCD* locus (spermine/putrescine ABC-transporter). The spermidine/putrescine transporter is involved in polyamine trafficking, which in turn plays a crucial role in DNA replication, cell division and stress response. Interestingly, polyamines have previously been implicated in the shift from a quiescent to a proliferating state in bacteria, plants, fungi and other eukaryotic systems [reviewed in 33,34]. Similarly, the *appABC* operon (peptide/nickel ABC-transporter) and the *ssuABC* operon (sulfonate/nitrate/taurine ABC-transporter) were up-regulated. The latter is of particular interest as taurine conjugated with cholate yields taurocholate, the primary germinant for *C. difficile* spores. One could speculate that the up-regulation of this transporter might be a part of a positive feedback loop providing a constant supply of the germinant and the basis for commitment in germinating spores. Other ABC-transporters that had at least one significantly up-regulated gene included maltose/maltodextrin ABC-transporter (9.5-fold), lactose/L-arabinose ABC-transporter (13.6-fold) and iron complex ABC-transporter (2.6-fold). In contrast, two ABC-transporter complexes involved in cobalt and nickel transport and encoded by the *cbiMNOQ* operon were down-regulated (Figure 4C).

The PTS system is a complex phosphate translocation mechanism involved in the transport of sugars such as glucose, mannose and mannitol. As such it plays a key role in cell metabolism driving glycolysis. Unlike ABC-transporters, sugar transport was largely inactive during germination with the entire branch of the system responsible for glucose trafficking significantly down-regulated (from 1.9- to 23.8-fold) in germinating spores when compared to vegetative cells, including members of the *pstG-ABC* operon. In contrast, a number of genes involved in fructose and lactose metabolism were up-regulated in germinating spores. These included *fruABC* (fructose-specific phosphotransferase; 75.2-fold), CD2270 (putative 1-phosphofructokinase; 79.8-fold) and CD1806 (putative fructokinase; 2.8-fold) (Figure 4D). While this could simply reflect the composition of the culture medium, it could also indicate the preference for fructose as the main carbon source in early germination.

### Ribosomal Proteins and Transcriptional Regulators

Exit from dormancy requires the bacterium to rebuild most of the structures found in a vegetative cell, necessitating large amounts of protein synthesis. It is not surprising then that a significant up-regulation of genes involved in transport, coincided with up-regulation of transcription and translation. In general, all genes encoding ribosomal proteins were up-regulated during germination. These included three genes encoding 30S subunit proteins and eight genes encoding 50S subunit proteins (Figure 4E). Similarly, genes encoding DNA-directed RNA polymerase were up-regulated, although only the genes encoding the β and β′ subunits of the polymerase had a *p* value ≤0.05. Furthermore, sixty-eight genes encoding transcriptional regulators were up-regulated (from 1.3- to 10.9-fold) while twenty-four genes were down-regulated (from 1.3- to 21.2-fold) in germinating spores (Figure 4F). These included members of the *gntR, tetR araC, marR, merR, rpiR, copR, deoR* and *lysR* gene families. On the whole, genes encoding tRNA synthetases were down regulated in germinating spores when compared to a vegetative cell. Similarly, enzymes involved in amino acid metabolism were largely down-regulated during germination, possibly reflecting the preferential catabolism of endogenous nutrients as a means for obtaining the necessary ‘building blocks’ for protein synthesis in early germination.

### Secretion and Cell Wall Components

The Sec machinery provides a major pathway of protein translocation from the cytosol across the cytoplasmic membrane in bacteria [35]. A number of components of the *sec* secretory pathway can be identified in *C. difficile 630* including *secA1, secA2, secE and secY* [27]. In general, all genes within the *secAYEG* operon were up-regulated in germinating spores, although only *secA2* (3.4-fold) and *secE* (3.8-fold) significantly so. The lipoprotein signal-peptidase *lspA* was also up-regulated (2.6-fold) as was the signal recognition particle encoded by *ffh* (4.16-fold) and prolipoprotein diacylglyceryl transferase encoded by *lgt* (3-fold).

The *C. difficile* flagellar assembly is encoded by thirty-five genes located within a single cluster. A vast majority of flagellar genes were inactive in germinating spores when compared to vegetative cells. In addition, five genes encoding components of the bacterial chemotaxis machinery including *motA, motB, cheY, cheD* and *cheW* were also found to be down-regulated in germinating spores (Table 2; Figure 4G) as were seven genes within two gene clusters encoding putative type IV pilus proteins (Table 3; Figure 4H). As cell motility is typically initiated upon nutrient deprivation in stationary phase, the overall inactivity of genes involved in motility during germination is in accordance with expectations, in that flagellar genes are dormant during germination and are transcribed in vegetative cells.

**Table 2:** List of differentially regulated flagellar assembly and chemotaxis genes.

Systematic name	Gene product	*p-*value	Fold change
CD0228	flagellar motor switch protein	0.0248	−1.9
CD0230	putative flagellar biosynthesis protein	0.0151	−3.7
CD0231	putative flagellar hook-associated protein	0.0204	−3.6
CD0232	flagellar hook-associated protein	0.0167	−5.0
CD0235	flagellar protein FliS	0.0106	−3.7
CD0236	flagellar protein	0.0161	−4.0
CD0237	flagellar cap protein	0.0165	−3.1
CD0239	flagellin subunit	0.0282	−2.0
CD0246	flagellar basal-body rod protein	0.0271	−3.1
CD0247	flagellar hook-basal body complex protein	0.0117	−3.8
CD0248	flagellar M-ring protein	0.0177	−3.9
CD0249	flagellar motor switch protein	0.0187	−4.8
CD0250	flagellar assembly protein	0.0115	−5.6
CD0251	flagellum-specific ATP synthase	0.0139	−5.7
CD0252	flagellar protein	0.0172	−6.1
CD0253	putative flagellar hook-length control protein	0.0177	−5.7
CD0255	flagellar hook protein	0.00833	−6.0
CD0255A	putative flagellar protein	0.0154	−6.2
CD0256	chemotaxis protein	0.0164	−6.4
CD0257	chemotaxis protein	0.0236	−6.0
CD0258	flagellar basal body-associated protein	0.00927	−5.3
CD0259	putative flagellar protein	0.0297	−4.9
CD0260	flagellar biosynthetic protein	0.0394	−4.4
CD0261	flagellar export protein	0.0393	−4.9
CD0262	flagellar export protein	0.0364	−5.4
CD0263	flagellar export protein	0.0232	−5.5
CD0265	flagellar number regulator	0.0284	−6.3
CD0268	flagellar basal-body rod protein FlgG	0.0078	−6.6
CD0269	putative flagellar basal-body rod protein	0.00932	−6.5
CD0270	putative flagellar motor switch protein	0.00953	−6.7
CD0271	putative flagellar motor switch protein	0.0475	−4.6
CD0533	chemotaxis protein	0.017	−1.8
CD0535	chemotaxis protein	0.0329	−1.9
CD0540	chemotaxis protein	0.0257	−1.6

**Table 3:** List of differentially regulated type IV pilin genes.

Systematic name	Gene product	*p-*value	Fold change
CD3295	putative type IV pilus-assembly protein	0.0211	+1.3
CD3504	putative type IV prepilin leader peptidase	0.00385	−2.4
CD3505	putative type IV pilus retraction protein	0.0186	−2.5
CD3507	putative type IV pilin	0.00579	−2.5
CD3508	putative type IV pilin	0.0141	−2.3
CD3509	putative type IV pilus assembly protein	0.0191	−2.3
CD3511	type IV pilus assembly protein	0.0245	−3.4
CD3512	type IV pilus assembly protein	0.0091	−2.9

As a hallmark of cell expansion during germination and outgrowth, genes involved in cell wall biosynthesis were found to be significantly up-regulated in germinating spores. Peptidoglycan is produced from N-acetylglucosamine (GluNAc) and N-acetylemuramic acid (MurNAc) in a series of reactions involving incorporation of D-glutamine and D-alanyl-D-alanine. Genes encoding the enzymatic components of this pathway are largely located within the *mur-mra* cluster. Ten enzymes involved in peptidoglycan biosynthesis were up-regulated in germinating spores, including *ddl* (D-Ala D-Ala ligase B; 7.3-fold) *glnA* (glutamine synthase; 2.6-fold) and *murF* (UDP-MurNAc-pentapeptide synthase; 2.4-fold) as well as other members of the *mur-mra* cluster: *murGDE* and *mraYW* (Table 4; Figure 4I). In addition to peptidoglycan, the Gram-positive cell wall can also contain secondary cell wall polymers (SCWP) such as teichoic, teichuronic acids and lipoteichoic acids which can make up between 10 and 60% of its structure. Although the genes specifying these components have not been identified in *C. difficile,* it might be relevant that a cluster of genes (CD2769-80) specifying glycotransferases and related enzymes was found to be significantly up-regulated in germinating spores (Table 5; Figure 4J).

**Table 4:** List of differentially regulated peptidoglycan biosynthesis genes.

Systematic name	Gene product	*p-*value	Fold change
CD0784	putative N-acetylmuramoyl-L-alanine amidase	0.0195	+2.0
CD1343	glutamine synthetase	0.0081	+2.6
CD1408	D-alanine–D-alanine ligase B	0.00843	+7.3
CD2651	UDP-N-acetylglucosamine–N-acetylmuramyl-(penta peptide) pyrophosphoryl-undecaprenol N-acetylglucosamine transferase	0.022	+1.4
CD2653	UDP-N-acetylmuramoylalanine–D-glutamate ligase	0.0105	+1.5
CD2654	phospho-N-acetylmuramoyl-pentapeptide-transferase	0.0145	+1.7
CD2655	UDP-N-acetylmuramoyl-tripeptide–D-alanyl-D-alan ine ligase	0.00508	+2.4
CD2664	putative UDP-N-acetylmuramoylalanyl-D-glutamate–2,6-diaminopimelate ligase	0.0344	+2.4
CD3563	putative spore cortex-lytic enzyme	0.0245	+2.2
CD1898	putative phage-related cell wall hydrolase (endolysin)	0.0174	−6.1

**Table 5:** List of differentially regulated putative secondary cell wall polymer (SCWP) biosynthesis genes.

Systematic name	Gene product	*p-*value	Fold change
CD2769	capsular polysaccharide biosynthesis protein	0.0201	+2.9
CD2770	putative capsular polysaccharide biosynthesis glycosyl transferase	0.00656	+2.7
CD2771	putative UDP-glucose 6-dehydrogenase	0.00681	+2.4
CD2772	putative teichuronic acid biosynthesis glycosyl transferase	0.016	+2.2
CD2773	putative beta-glycosyltransferase	0.0115	+2.3
CD2774	putative teichuronic acid biosynthesis glycosyl transferase	0.019	+2.2
CD2775	putative minor teichoic acid biosynthesis protein	0.016	+2.3
CD2776	putative glycosyl transferase	0.0408	+2.4
CD2777	putative polysaccharide polymerase	0.0182	+2.5
CD2778	putative polysaccharide biosynthesis protein	0.0311	+2.5
CD2779	putative mannose-1-phosphate guanylyltransferase	0.0116	+2.2
CD2780	putative phosphomannomutase/phosphoglycerate mutase	0.00636	+2.7

### Toxins

Of central importance in *C. difficile* is the pathogenicity locus (PaLoc) containing five genes encoding two large clostridial toxins: TcdA and TcdB as well as their regulators (*tcdC, tcdR*) and export machinery (*tcdE*) [36]. Both toxin A and B are represented by multiple probes on the microarray. *tcdA* was down-regulated during germination when compared to the vegetative cell. No transcripts for *tcdB* could be detected in either state, consistent with the observation that toxins are expressed in stationary phase.

### Concluding Remarks

This study aims to give a genome-wide overview of the temporal gene expression during germination and outgrowth of *C. difficile* spores. We decided to focus on the events that follow initiation of germination and are critical in the transformation of a metabolically dormant spore into an actively growing vegetative cell. Our analysis was possible as we initially demonstrated the synchronous nature of germination in *C. difficile*. This is intriguing as germination in *Bacillus* species and even the more closely related *C. perfringens* and *C. botulinum* has been shown to be heterogenous [37]–[40]. While the vast majority of a spore population will enter germination upon exposure to germinants, a small proportion may not germinate for many hours or even days. This ‘superdormancy’ has been correlated with a low level of specific germination receptors in individual spores [41] and is thought to be an example of ‘bet hedging’, ensuring the survival of a given population in a rapidly changing environment. Under the conditions tested, 99.9999% of the *C. difficile* 630 spore population germinated synchronously. This might be a reflection of the unique germination mechanism present in *C. difficile* and/or the fact that the human gut is a relatively stable environment eliminating the need for super-dormancy.

Our results will give insight into how a dormant organism re-establishes its metabolism, re-builds the basic structures of the vegetative cell and resumes growth. Around 14% of genes (511 genes out of 3679) were found to be significantly differentially regulated at 30 min of germination when compared to the vegetative cell and a number of functional groups of genes appeared to be co-regulated. Further analysis of the genes and biochemical pathways identified here as important in germination will enable a more targeted investigation of germination in *C. difficile* endospores.

## Experimental Procedures

### Bacterial Strains and Culture Conditions

*C. difficile* 630 (*tcdA*+ *tcdB*+; epidemic strain isolated in 1985 from Zurich, Switzerland; PCR ribotype 012) was routinely cultured on blood agar base II (Oxoid) supplemented with 7% horse blood (TCS Biosciences), brain-heart infusion (BHI) agar (Oxoid) or in BHI broth (Oxoid). Cultures were grown statically in an anaerobic cabinet (Don Whitley Scientific) at 37°C in an anaerobic atmosphere (10% CO_2_, 10% H_2_ and 80% N_2_). The strain has been fully sequenced [15] and its genome sequence is available at http://www.sanger.ac.uk/resources/downloads/bacteria/clostridium-difficile.html).

### Genomic DNA Extraction

*C. difficile* 630 was collected from 10 ml late-log-phase culture by centrifugation at 5,000×g for 10 min. Genomic DNA was purified through subsequent incubations with lysozyme (1 h at 37°C), pronase (1 h at 55°C), 10% N-lauroylsarcosine (1 h at 37°C) and RNase (1 h at 37°C) followed by phenol/chloroform extraction and ethanol precipitation.

### Sporulation

*C. difficile* 630 sporulation was induced as described previously [42]. Briefly, 10 ml of TGY broth (3% tryptic soy broth; 2% glucose; 1% yeast extract; 0.1% L-cysteine) was inoculated with a single colony of *C. difficile* 630 grown on BHIS agar (brain heart infusion agar supplemented with 0.1% L-cysteine and 5 mg/ml yeast extract). Following over-night static incubation, bacteria were sub-cultured 1∶10 in SMC broth (9% Bacto peptone, 0.5% proteose peptone, 0.15% Tris base, 0.1% ammonium sulphate), incubated until the culture reached OD_600_ of 0.6 and spread out on SMC agar plates. After 7 days of anaerobic incubation at 37°C, spores were harvested in 2 ml of ice-cold sterile water.

### Spore Purification

Crude spore suspensions were washed five times with ice-cold sterile water and vortexed for 10 min in between washes. The resulting pellets were re-suspended in 500 µl of 20% HistoDenz (Sigma) and layered over 1 ml of 50% HistoDenz in a 1.5 ml tube. Tubes were centrifuged at 14,000×g for 15 min. The spore pellet was recovered and washed three times with ice-cold sterile water to remove residual HistoDenz. Spore purity was assessed *via* phase contrast microscopy. Spore yields in individual preparations were estimated by counting colony forming units (CFU) on BHI agar plates supplemented with 0.1% sodium taurocholate (Tch). Purified spores were stored in water at 4°C until further analysis.

### Germination Assay

Purified *C. difficile* 630 spores were re-suspended to and OD_600_ of 0.1 in BHIS ±0.5% Tch and incubated under aerobic or anaerobic conditions for 6 h at 37°C. Growth was monitored *via* OD_600_ measurements. CFU enumeration was carried out by plating 10-fold dilutions of the germinating cultures on BHIS +0.1% Tch. For spore enumeration, samples were heated at 70°C for 30 min prior to plating.

### Phase Contrast and Fluorescence Microscopy

Samples were spun down (5,000×g; 10 min; 4°C), washed with 1 ml of PBS and fixed with 3.7% formaldehyde for 15 min at RT. Following fixation, samples were washed with PBS once more and dried onto glass slides. DNA staining was carried out by adding Hoechst 33258 dye to the mounting medium to a final concentration of 20 µg/ml. Phase contrast and fluorescence microscopy were carried out according to standard procedures on an Eclipse E600 microscope (Nikon) using a 100× oil immersion lens. Images were captured using a Retiga-400R Charge Coupled Device (Q-Imaging).

### RNA Extraction and RT-PCR

RNA was extracted using the FastRNA Pro Blue Kit (MP Biosciences). Briefly, 5 ml cultures containing 5×10^9^ endospores (OD_600_ 10) at various stages of germination were mixed with 10 ml of RNA Protect (Qiagen) and incubated for 5 min at RT. Samples were centrifuged (5,000×g; 10 min; 4°C), resuspended in 1 ml of RNA Pro solution and transferred to a lysing matrix tube containing 0.1 mm silica beads. Tubes were processed in a FastPrep-24 instrument (MP Biosciences) (3×45 s at 6.5 ms^−1^ with 2 min of cooling on ice between cycles). The efficiency of spore rupture was evaluated *via* phase contrast microscopy as well as by plating spore samples prior to and post processing, indicating that only 0.0001% of the spore population remains intact. Following disruption, samples were centrifuged to remove spore debris and silica beads from suspension (16,000×g; 10 min; 4°C). Approx. 700 µl of liquid was transferred to an RNase-free tube and incubated at RT for 5 min. 300 µl of chloroform was added. The sample was vortexed for 10 s and centrifuged (16,000×g; 15 min; 4°C) to separate the phases. The aqueous phase was transferred to a fresh tube containing 200 µl of 95% EtOH and placed on ice. The mixture was transferred to a spin column assembly (SV Total RNA isolation system; Promega) and centrifuged (16,000×g; 1 min). Columns were washed twice with 600 µl and 250 µl of RNA wash solution. RNA was eluted in 45 µl of RNase-free water. DNase treatment was carried out using the Turbo DNase kit (Ambion) according to manufacturers protocol. The enzymatic reaction was cleaned-up using the RNeasy Mini Kit (Qiagen) RNA purity and quantity was determined by nanodrop UV spectroscopy. RNA integrity was confirmed on a RNA 6000 nano lab-Chip using a Bioanalyzer 2100 instrument (Agilent). Samples were stored at −80°C until further analysis.

### Reverse Transcriptase PCR

For conventional RT-PCR, first strand cDNA was synthesized from total RNA using random decamers as primers and the RETROscript Kit (Ambion) according to manufacturer’s instructions and then used in standard Taq (Sigma) PCR reactions using gene specific primers (Table S3).

### Microarray Analysis

The microarray was constructed by determining all unique genes from the predicted coding sequences of *C. difficile* strains 630, QCD-32g58, 196, R20291 plasmid pCD630. Multiple optimal hybridisation 60-mer oligonucleotide sequences were designed (Oxford Gene Technologies), from which a minimal non-redundant subset of oligonucleotides were selected with a target coverage of three 60-mers per gene. Arrays were manufactured on the Inkjet *in situ* synthesized platform (Agilent) using the 8×15 k format.

Competitive genomic DNA/RNA hybridizations were carried out according to standard Agilent protocols. Briefly, 5 µg of total RNA was labelled with Cy3 *via* reverse transcription using SuperScript II (Invitrogen). 1 µg of gDNA was labelled with Cy5 using Exo-Klenow fragment. Both labelling reactions were cleaned-up using the PCR Purification MiniElute Kit (Qiagen). Cy3-labeled cDNA and Cy5-labeled gDNA were mixed with 10× blocking agent and hybridization buffer and incubated for 3 min at 95°C and 30 min at 37°C. Samples were then applied to a *C. difficile* OGT array CDv2.0.1 (BµG@S) and incubated for 24 h in a hybridization oven set to 65°C. The microarray slide was washed, fixed in acetonitryl, dried and scanned using an Agilent G2565CA Scanner. Microarray data extraction was performed using ImaGene software (BioDiscovery), and further processed using MAVI Pro software (MWG Biotech). Normalization and statistical analysis were performed using GeneSpring v7.3.1 software (Agilent Technologies). Briefly, gene specific data was derived from average intensity of between 1 and 5 oligonucleotide reporters. Gene values below 0.01 were set to 0.01. Each gene’s measured intensity was divided by its control channel value in each sample; if the control channel was below 10 then 10 was used instead. If the control channel and the signal channel were both below 10 then no data was reported. Each measurement was divided by the 50th percentile of all measurements in that sample. Replicate time points were normalized to the 180 minute time point: each measurement for each gene was divided by the median of that gene’s measurements in the corresponding control samples. Following the initial experiment covering eight time points (0′, 15′, 30′, 45′, 60′, 90′, 120′ and 180′), two additional biological replicates were carried out for time points 30′, 60′, 90′ and 180′. Each time point was independently tested versus 180 min using 1-way ANOVA using Benjamini-Hochberg multiple testing correction and p = 0.01 or 0.05.

### Accession Numbers

The array design (CDv2.0.1) is deposited in BµG@Sbase (Accession No. A-BUGS-49; http://bugs.sgul.ac.uk/A-BUGS-49) and ArrayExpress (Accession No. A-BUGS-49). Fully annotated microarray data have been deposited in BµG@Sbase (accession number E-BUGS-145; http://bugs.sgul.ac.uk/E-BUGS-145) and also ArrayExpress (accession number E-BUGS-145).

## Supporting Information

Figure S1 **RNA quality control.** A) Gradual increase in RNA yield observed during spore germination. B) Bioanalyzer pseudogel with RNA Integrity (RIN) values C) Bioanalyzer electropherograms showing two distinct peaks corresponding to 16S and 23S rRNA. In addition, two smaller rRNA species were identified in dormant spores and in early germination, represented by small peaks on Bioanalyzer spectra. 5S rRNA peak visible at retention time 23 seconds.
(DOCX)

Table S1 **List of genes up and down regulated during germination.**
(XLS)

Table S2 **Abundant spore transcripts.**
(XLS)

Table S3 **Primers used in this study.**
(DOCX)
