# Supplementary figures and images for: Transcriptional Analysis of Temporal Gene Expression in Germinating Clostridium difficile 630 Endospores
Source: PLoS One. 2013 May 15;8(5):e64011. doi: 10.1371/journal.pone.0064011 (PMC3655068; doi:10.1371/journal.pone.0064011)

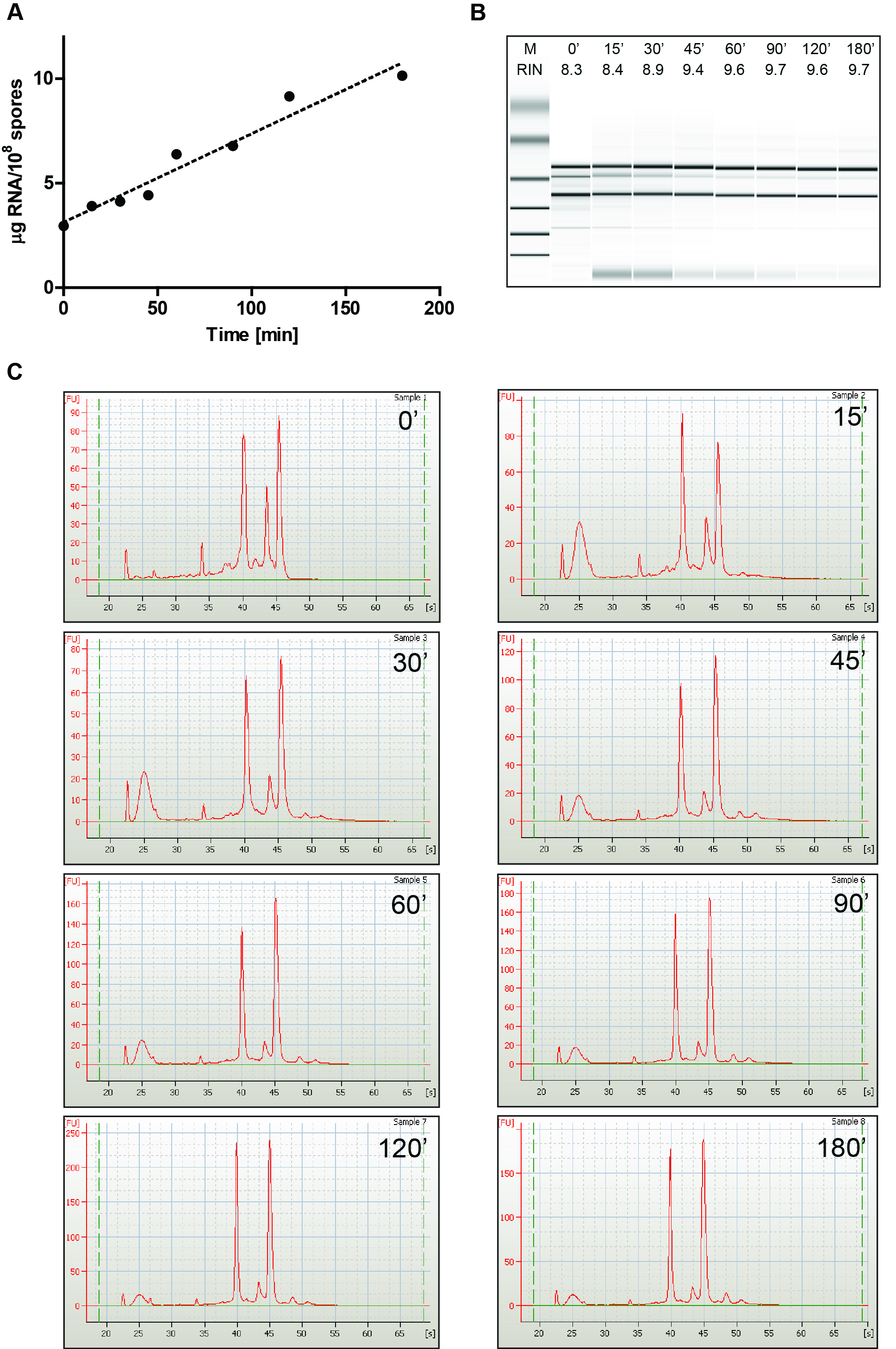
Figure S1.

Supplement: Figure S1 — RNA quality control. A) Gradual increase in RNA yield observed during spore germination. B) Bioanalyzer pseudogel with RNA Integrity (RIN) values C) Bioanalyzer electropherograms showing two distinct peaks corresponding to 16S and 23S rRNA. In addition, two smaller rRNA species were identified in dormant spores and in early germination, represented by small peaks on Bioanalyzer spectra. 5S rRNA peak visible at retention time 23 seconds. (DOCX) [file pone.0064011.s001.docx]
