# Supplementary material for: Transcriptional Analysis of Temporal Gene Expression in Germinating Clostridium difficile 630 Endospores
Source: PLoS One. 2013 May 15;8(5):e64011. doi: 10.1371/journal.pone.0064011 (PMC3655068; doi:10.1371/journal.pone.0064011)
Supplement: Table S3 — Primers used in this study. (DOCX) [file pone.0064011.s004.docx]

| **Gene target** | **Official name** | **Orientation** | **Sequence** |
| --- | --- | --- | --- |
| CD2782 | NF1671 | Forward | TCCCTCTTATTCTTTCTACTTTCATTCC |
|  | NF1672 | Reverse | GAAGGGAGTATACCAGAGATTTCG |
| CD2796 | NF1673 | Forward | GCTGGTTTATCACTTGTTCCTTTTCC |
|  | NF1674 | Reverse | TTCATTTCAAGCCAGTATAAAAGTACAAGG |
| CD0663 | NF1677 | Forward | CCATCAAACAATGTAGAAGAAGCTGG |
|  | NF1678 | Reverse | TAGCAAATTCGCTTGTGTTGAATTCATC |
| CD0660 | NF1679 | Forward | AGATTTATGATGGAACTAGGAAAGTATTTAAG |
|  | NF1680 | Reverse | CACCAAGAGAACCTTCAAAATAATTCC |
| CD2625 | NF1681 | Forward | TCTAGCAAACGCTGCATGTGC |
|  | NF1682 | Reverse | ACAACCACTAAATCCACTCCAGG |
